# Patient safety: Needs and initiatives

**DOI:** 10.4103/0972-5229.42559

**Published:** 2008

**Authors:** Julian Bion

**Affiliations:** **From:** Department of Intensive Care Medicine, University of Birmingham, UK

**Keywords:** Patient safety, best practice, quality, medical errors, teamwork

## Abstract

Patient safety has become a major defining issue for healthcare at the beginning of the 21^st^ century. Viewed from the perspective of reliability of delivery of best practice, healthcare systems demonstrate a degree of imperfection which would not be tolerated in industry. In part, this is because of uncertainty about what constitutes best practice, combined with complex interventions in complex systems. The acutely ill patient is particularly challenging, and as the majority of admissions to hospitals are emergencies, it makes sense to focus on this group as a coherent entity. Changing clinical behavior is central to improving safety, and this requires a systems-wide approach integrating care throughout patient journey, combined with incorporating reliability training in life-long learning.

## Introduction

In 2000, the Institute of Medicine's report ‘To Err is Human’[1] estimated that as many as 98,000 patients could die from avoidable mistakes each year in the USA alone, with many more suffering complications and varying degrees of morbidity. The editors and contributors challenged healthcare systems in the USA and worldwide to focus our efforts on reducing harm to patients caused by errors in healthcare delivery. Since then many countries have established national patient safety organizations, with some coordination of effort through the World Health Organization's World Alliance for Patient Safety.[2] A Medline search reveals that between 2000 and May 2008, 29 566 articles have been published on aspects of patient safety - and this excludes a further 16 788 focused on quality assurance. There is now general recognition that error in healthcare is common, and that patients suffer avoidable harm as a result.

## Methodological Considerations

However, less certain is the nature of the linkage - how and to what extent error actually contributes to adverse longterm outcomes - and how to demonstrate that improvements in processes of care result in improvements in outcome. Research methodology is central to unraveling these doubts and uncertainties. The first problem is how one identifies errors and links them to outcomes. For example, Hofer and colleagues have shown[3] that while it may be possible to identify errors using expert case note review, and to associate those errors with a shortterm adverse event, it is much more difficult to be certain that the error contributed to adverse longterm outcomes, for which the main determinant is usually the patient's underlying disease. Case note review is the standard method for retrospective audit, but there are substantial differences in the way in which doctors and nurses interpret processes and outcomes of care.[4] These discrepancies can be reduced by using explicit criterion-based audit rather than implicit ‘expert review’, provided that there is a strong evidence base for the criteria selected as the gold standard.[5] The problem is that for many diseases (particularly emergency care) the evidence base may not be strong, and where standards of care exist they may not fully reflect the complexity of potential treatment pathways, or may lack solid professional endorsement.

The second problem is related to the context in which error may arise. To characterize a problem or determine the efficacy of an intervention, it is necessary to express these variables as ‘rates’ - that is, as numerator and denominator. In patient safety research, this means knowing the number of errors in relation to the number of opportunities for error in order to determine the prevalence. Complex acute care environments are likely to have more opportunities for error, as well as better monitoring and hence detection of errors when they occur. Measuring error frequency alone will give a false impression of the scale and the nature of the problem.

The third problem is in trial design. Virtually all studies of interventions to improve patient safety employ a before-and-after design, because in quality improvement research it is either unethical to omit best practice in a control group, or difficult to prevent ‘contamination’ between the intervention and control groups (often referred to as the Hawthorne effect). Moreover, in complex systems there are many potential confounders. Cluster randomization has been used to minimize some of these problems, but the difficulty of adequate sample size persists. Stepped cluster randomization[6] may be advantageous, as this permits all centers to participate in both the control and the intervention arms. Without some form of control, it will not be possible to attribute with confidence the effect of a health services intervention unless the effect size is very large, as was the case for a study demonstrating substantial reductions in central venous catheter-related blood stream infections.[7] It has not been possible to achieve this for complex interventions such as medical emergency teams (rapid response teams or outreach care), where improvements in outcome were equivalent between the intervention and non-intervention hospitals,[8,9] thus perhaps reflecting systems-wide changes. We need to become much more sophisticated in arguing for funding for quality improvement research, and to specify the precise content of complex interventions rather than just evaluating the vehicle.[10]

## The Acutely III Patient: A Systems-wide challenge

At the same time that healthcare systems worldwide are trying to improve patient safety, they also have to deal with a growing emergency workload while contending with cost containment. This makes the acutely ill patient a prime target for safety initiatives, particularly because these patients have a high risk of morbidity and mortality, and are particularly susceptible to healthcare error.[11] The problem is that few specialities regard the care of acutely ill patients as their main core business, being more focused on (and interested in) elective workload and predictable funding streams. As a result, emergency admissions and acutely ill patients in general could substantially impair the efficacy of national and international patient safety initiatives,[12] because of the special challenges they bring in terms of process control, multidisciplinary teamwork teamwork, and translation of research evidence into best practice guidelines requiring implementation across specialities and geographical areas. Best practice needs to become engrained in competency-based training, from undergraduate to specialist level.[13] These objectives require transdisciplinary national and international collaboration.

## The Size of the Problem

Care of the acutely ill patient is a major activity for all health care systems. The proportion of patients who are admitted as emergencies varies between health care systems, and although precise figures are not available for many countries, they are likely to be between 30% (USA)[14] to over 60% (UK)[15,16] or between 99 (Canada) to 205 (Germany) admissions per 1000 population (OECD).[17] These figures do not include additional numbers of patients who deteriorate while in hospital undergoing elective treatment. The number of emergency hospital admissions is also increasing worldwide.[18] Acutely ill patients are therefore a major part of the business of healthcare systems. As populations age and healthcare becomes more complex, patient dependency increases; simultaneous reductions in the number of acute hospital beds and increases in throughput place additional pressures on the system, thereby increasing opportunities for error. Hospitals will therefore need to evolve new strategies for improving patient safety focused at least in part on this large population of the acutely ill.

## Responsibility and Recognition

Responsibility for the initial management of acutely ill patients in many hospital systems is often consigned to more junior staff, particularly at night or weekends. Although virtually all medical disciplines are responsible for acutely ill patients, they are not generally their ‘core business’, since most specialities tend to focus on out-patient care and elective interventions. Outside the core specialities of emergency medicine and critical care, many senior staff loose expertise in acute care management. Thus, the early warning signs of worsening acute illness are often overlooked, and patients may deteriorate to the point of cardiopulmonary arrest. Acutely ill patients are usually a source of considerable anxiety for junior medical and nursing staff, who welcome better training in acute care and support to enable them to provide more effective (i.e.: safer) care to unstable patients. Hospitals need to invest in providing both staff and training.[19]

## Integration, Teamwork and Decision Support

Constraints on working hours and difficulties with adequate staffing requires health care systems to develop different ways of providing emergency care. Gaps and discontinuities are common causes of error and miscommunication[20] and communication failures are a common cause of discontent amongst patients and relatives.[21] Transdisciplinary teamworking teamwork is generally presented as the solution and aviation crew resource management as the model, but substantial investment in training and education will be needed to make this a reality. Acute care provides an ideal testing ground for team building, an appropriate analogy being the military, not civilian aviation. Electronic systems which integrate clinical and laboratory information with prescribing can substantially reduce errors, but their true potential requires the inclusion of clinical decision support - prompts and reminders which enforce and facilitate best practice. Hospital design can similarly promote or impede patient safety.[22]

## Process Control: A Key Element in Acute Care

Twenty or more years ago it was common for patients with diabetic ketoacidosis, asthma or myocardial infarction to receive suboptimal treatment and to require admission to intensive care. Such patients now receive better care both in the community, and in hospital through more prompt and effective protocol-driven therapy. In consequence, the great majority can be treated in emergency departments and discharged home without requiring hospital admission, or in wards and specialized units instead of being admitted to intensive care. This transformation has been slow, but has come about through improved process control - better application of current knowledge. By contrast, process control is poor for the generality of acutely ill patients, particularly those who deteriorate after hospital admission. The Surviving Sepsis Campaign[23] provides an example of what can be achieved by focusing on a specific but poorly characterized entity, sepsis. A similar approach is now required for all acutely ill patients at risk of critical illness.

## Futile Care is Bad Care

As populations age, so does the hospital population. The elderly are more susceptible to adverse events and error[24] and are also less able to withstand the consequences. Ageing populations have more chronic and comorbid disease, which in the end will present as an acute deterioration. Providing appropriate care to these patients requires not only an understanding of the ethical issues involved, but a practical understanding of what is possible in acute care. The public as well as health care professionals need to develop a better understanding of the likely outcomes from interventions intended to save lives, in order to avoid imposing burdensome and futile care with consequential waste of limited health care resources. Early recognition of high risk patients permits earlier intervention which can either result in treatment to prevent in-hospital cardiac arrest[25] or allows time for discussion about treatment limitation so that an inevitable death can occur peacefully, untroubled by useless technology.

Patient safety: The reliable implementation of best practice care

The key to greater safety is to improve the reliability of delivery of best practice. Until recently it was assumed that all that was required was high quality research demonstrating the superiority of one intervention or treatment over another. We now know that this is insufficient, and that failures of translation result in medical care that is not reliable.[26–28] What we do not know is why this should be so. What are the barriers to implementing best practice? Are they generic, or are there context-specific barriers as well? Reliability of care can be improved, but the effort required is considerable[29] and a formal strategy is needed to implement and sustain improvements in processes of care. Changing clinician behavior is a complex intervention, requiring leadership at multiple levels within an organization, and incorporation of best practice in life-long learning.[30–31] That in turn means that the professions have to develop methods for evaluating current evidence, work out what to do in the presence of uncertainty, and apply a greater degree of standardization in our practice than we have previously been willing to accept. While some clinicians may regard this as a loss of clinical freedom, for our patients it may make the difference between life and death.
